# Recurrence of hepatocellular carcinoma in patients with high HALP score in TACE combined with ablation

**DOI:** 10.3389/fonc.2025.1609260

**Published:** 2025-09-03

**Authors:** Da Fang, Xue Yin, Xiaoyan Ding, Jinglong Chen, Xiongwei Cui, Caixia Hu

**Affiliations:** ^1^ Beijing Youan Hospital, Capital Medical University, Beijing, China; ^2^ Department of Cancer Center, Beijing Ditan Hospital, Capital Medical University, Beijing, China; ^3^ Department of Infectious Disease, The Third Xiangya Hospital, Central South University, Changsha, Hunan, China

**Keywords:** hepatocellular carcinoma, HALP score, Lasso-Cox regression, recurrence, nomogram

## Abstract

**Objectives:**

To investigate the relationship between the HALP score and recurrence in hepatocellular carcinoma (HCC) patients treated with transarterial chemoembolization (TACE) and ablation.

**Methods:**

We collected clinical data from 728 HCC patients who underwent TACE and ablation from January 2018 to December 2023. Patients with high HALP scores (H-HALP, n=422) were stratified into a training cohort (n=296) and an internal validation cohort (n=126), while an external validation cohort (n=147) was independently enrolled. Lasso-Cox regression was employed to identify independent risk factors for recurrence-free survival (RFS), and a nomogram was constructed. The predictive accuracy of nomogram was evaluated using receiver operating characteristic (ROC) curves, calibration curves, and decision curve analysis (DCA).

**Results:**

Although the median RFS in the H-HALP group longer than the L-HALP group (1.84 vs. 1.60 years, *P*=0.024), recurrence rates remained substantial in H-HALP patients (1-/3-/5-year RFS: 70.8%/36.2%/21.5%). The nomogram, integrating cirrhosis, tumor numbers, and γ-glutamyl transpeptidase (GGT), demonstrated moderate predictive accuracy for 1-/3-/5-year RFS in the training cohort (AUC: 0.665/0.694/0.671) and internal validation cohort (AUC: 0.622/0.606/0.561). External validation yielded AUCs of 0.569 (1-year), 0.615 (3-year), and 0.662 (4-year). Calibration curves indicated strong agreement between predicted and observed outcomes, while DCA confirmed clinical utility. Risk stratification based on nomogram scores revealed significantly prolonged RFS in low-risk versus high-risk groups across all cohorts.

**Conclusion:**

The HALP score alone showed limited prognostic value in this cohort; however, the Lasso-Cox regression-based nomogram effectively stratified recurrence risk in H-HALP patients treated with TACE and ablation.

## Introduction

Hepatocellular carcinoma (HCC) is the fifth leading cause of cancer and the fourth leading cause of cancer-related mortality globally, characterized by high rate of recurrence and metastasis (1, 2). For early-stage HCC, surgical resection, liver transplantation and local ablation are considered potentially curative therapy (3). However, only approximately 20% of HCC patients derive a survival benefit from resection and transplantation (4). Locoregional therapies, particularly transarterial chemoembolization (TACE) and ablation, play a leading part in the management of 50-60% of HCC cases (5).

TACE, a minimally invasive procedure, induces tumor ischemia by selectively delivering chemotherapeutic agents and embolic materials to tumor-feeding arteries, effectively downstaging lesions and reducing tumor burden (6). Nonetheless, incomplete embolization and residual microvascular invasion often lead to recurrence (7). Conversely, ablation techniques (e.g., radiofrequency or microwave ablation) achieve localized tumor destruction but face limitations in treating large (>3 cm) or perivascular tumors due to heat-sink effects (5). The synergistic combination of TACE and ablation addresses these shortcomings. TACE reduces tumor vascularity, enhancing thermal ablation efficacy, while ablation eradicates residual lesions post-TACE (8). Previous studies have confirmed that this combined approach significantly prolongs recurrence-free survival (RFS) compared to monotherapy (4, 9, 10). Despite these advancements, recurrence and distant metastasis remain seriously affecting the overall survival of HCC patients. Therefore, early identification and prompt treatment of individuals at high risk of recurrence is essential to improve outcomes for patients with HCC.

Nutrition and systemic inflammatory responses are associated with tumor efficacy and survival. The HALP score, defined as hemoglobin (Hb) × albumin (ALB) × lymphocytes (LYM)/platelets (PLT), comprehensively evaluate the inflammatory response and nutritional status (11, 12). It has been demonstrated as an effective prognostic predictor in various solid cancers, such as gastric carcinoma, colorectal cancer, and renal cell cancer (13–15). In HCC, a low HALP score is associated with poor prognosis in patients undergoing liver resection and is predictive of postoperative recurrence (16–18). However, its predictive value in patients undergoing TACE-ablation remains unexplored.

This study aims to evaluate the association between HALP scores and recurrence in HCC patients receiving combined ablation and TACE. Additionally, we seek to develop a nomogram to improve the individualized prediction of recurrence risk in this populations, which may identify the high-risk patients in advance to implement effective preventive measures.

## Methods and materials

### Patients selection

This study retrospectively enrolled 728 HCC patients who received TACE combined ablation at Beijing Youan Hospital, Capital Medical University, between January 2018 and December 2023. HCC diagnosed by histological or radiological criteria as defined by the American Association for the Study of Liver Diseases (AASLD) guidelines (19). Patients were stratified into two groups based on the HALP score cutoff value (-56.8), as established in prior research (15): 422 patients with high HALP scores (> -56.8, H-HALP group) and 306 patients with low HALP scores (≤ -56.8, L-HALP group). The H-HALP cohort was further randomly divided into a training cohort (n=296) and an internal validation cohort (n=126) at a 7:3 ratio, with a randomization seed set at 400. Furthermore, an independent external validation cohort comprising 147 H-HALP patients treated at Beijing Ditan Hospital during the same period was included to test the model’s robustness. Inclusion criteria were as follows: (1) aged ≥ 18 years; (2) BCLC stage 0, A or B; (3) Child-Pugh class A or B liver function; (4) patients with tolerable general status: Eastern Cooperative Oncology Group (ECOG) performance status of 0–2 and stable organ function adequate for interventional therapy; (5) underwent TACE followed by ablation, with radiological confirmation of complete ablation, defined as the absence of contrast enhancement in the treated lesion on follow-up imaging one month after the procedure. Exclusion criteria included: (1) received systemic drugs before TACE combined with ablation, including sorafenib, lenvatinib, PD-1 inhibitors, etc. (2) presence of other primary malignant tumors. (3) lost to follow-up. (4) contraindication to TACE or ablation.

This study was conducted by the Declaration of Helsinki, and experienced clinicians determined patient eligibility for combined therapy based on guidelines. In addition, this study was approved by the Ethics Committee of Beijing Youan Hospital, Capital Medical University, and informed consent from the patients was waived due to its retrospective nature. All patient data were de-identified to protect privacy.

### Variable collection

Demographic and clinicopathological data were retrospectively collected for analyzed. Baseline characteristics included age, gender, tumor size, tumor number, hypertension, diabetes, cirrhosis, antiviral treatment, BCLC stage and Child-Pugh classification. Laboratory values were obtained from the closest test performed within 7 days before treatment. If multiple measurements were available, the most recent value prior to treatment initiation was used for analysis. Laboratory parameters included Hb, red blood cell (RBC) count, white blood cell (WBC) count, ALB, LYM count, PLT count, alanine transaminase (ALT), aspartate aminotransferase (AST), total bilirubin (TBIL), direct bilirubin (DBIL), γ-glutamyl transpeptidase (GGT), globulin (Glob) and alpha-fetoprotein (AFP). The HALP score was calculated as Hb (g/L) × ALB (g/L) × LYM (10^9^/L)/PLT (10^9^/L).

### Therapeutic procedure

All patients underwent conventional TACE (cTACE) performed by two interventional radiologists with over five years of experience. The procedure was carried out under local anesthesia using the Seldinger technique via femoral artery access. Tumor-feeding arteries were identified through digital subtraction angiography (DSA). A chemotherapeutic emulsion consisting of 20 mg of epirubicin mixed with 6–10 mL of lipiodol was selectively injected into the tumor-feeding vessels. This was followed by embolization using gelatin sponge particles until stasis of blood flow was achieved. If any adverse reactions occurred during the procedure, symptomatic treatment was administered accordingly.

Ablation was conducted within two weeks post-TACE, utilizing radiofrequency ablation (RFA; Cool-tip RF Ablation System, Covidien, USA) and microwave ablation (MWA; ECO Microwave Ablation System, China). Among all patients, 312 (42.9%) received RFA and 416 (57.1%) received MWA. The ablation range completely covered the tumor to the edge of 0.5-1.0 cm to prevent marginal residue and recurrence. For MWA, a typical power setting of 40 to 60 watts was used, with an average ablation time of approximately 5 minutes per site. For more aggressive or multi-focal lesions requiring repeated ablation, total energy application ranged accordingly. In RFA procedures using emission-frequency modes, higher power outputs (120–160 watts) were employed, with each ablation site treated for 6–8 minutes. The selection of power and duration was individualized based on tumor location, size, and surrounding structures. The ablation protocol comprised the following steps: (1) determining the appropriate ablation position using contrast-enhanced computed tomography (CT) or magnetic resonance imaging (MRI); for MRI, a 1.5T system with T1-weighted dynamic contrast-enhanced sequences and T2-weighted imaging was used to assess tumor extent and vascular involvement. (2) inserting the ablation needle at the marked site and monitoring the procedure via imaging; (3) expanding the ablation area as necessary, considering multiple sites and potential overlapping or repeated ablation; (4) heating the needle track in the final phase to prevent tumor implantation and postoperative bleeding; and (5) conducting post-ablation imaging to evaluate treatment efficacy and complications, with follow-up contrast-enhanced CT or MRI performed one-month post-procedure.

### Follow-up

All patients were followed up every three months during the first year and every six months thereafter in the outpatient clinic, in accordance with the Chinese Guidelines for Primary Liver Cancer (2024 edition) (20) and the AASLD guidelines (19). Follow-up assessments included liver function tests, routine blood tests, serum alpha-fetoprotein (AFP) levels, and imaging examinations (contrast-enhanced CT or MRI).

The final follow-up date was December 31, 2023. The median follow-up time was 25.1 months. Radiographic recurrence was defined as the appearance of new intrahepatic or extrahepatic enhancing lesions consistent with viable tumor, based on the modified Response Evaluation Criteria in Solid Tumors (mRECIST) (21). Recurrence-free survival (RFS) was calculated from the date of initial treatment to the date of radiologic diagnosis of recurrence.

### Statistical analysis

All statistical analysis were performed using R software (version 4.1.3, http://www.rproject.org), with a two-sided *P* < 0.05 considered statistically significant. Demographic and clinical characteristics were compared among the training, internal validation and external validation cohorts. Continuous variables were presented as mean ± standard deviation and compared using the Student’s t-test or Mann-Whitney U test, depending on data distribution. Categorical variables were described as frequencies (percentages) and compared by Chi-square test. RFS was estimated using the Kaplan-Meier method, and differences between groups were assessed by the log-rank test. Independent predictive factors of RFS were evaluated using univariate and multivariate Cox proportional hazards models, incorporating variables with *P* < 0.05 from the univariate analysis into the multivariate analysis. Furthermore, LASSO-Cox regression was performed in the training cohort to identify independent prognostic factors for RFS, which were used to construct a nomogram. The predictive performance of the nomogram was evaluated using the area under the receiver operating characteristic (ROC) curve (AUC). Additionally, the nomogram’s predictive accuracy was also compared with the latest version of the American Joint Committee on Cancer (AJCC) TNM staging system and the factors (cirrhosis, tumor number and GGT) used to construct the nomogram. Calibration curves and decision curve analysis (DCA) were employed to assess the model’s calibration accuracy and clinical utility, respectively. External validation was conducted using an independent cohort from Beijing Ditan Hospital. The established prediction model was applied to the external data to calculate individual risk scores, and model performance was evaluated as described above. Patients in each cohort (training, internal validation, and external validation) were independently stratified into high- and low-risk groups based on the median risk score within the respective cohort. Kaplan-Meier analysis and the log-rank test were conducted separately in each cohort to compare RFS between risk groups.

## Result

### Survival analysis of the HALP score

**Figure 1:**
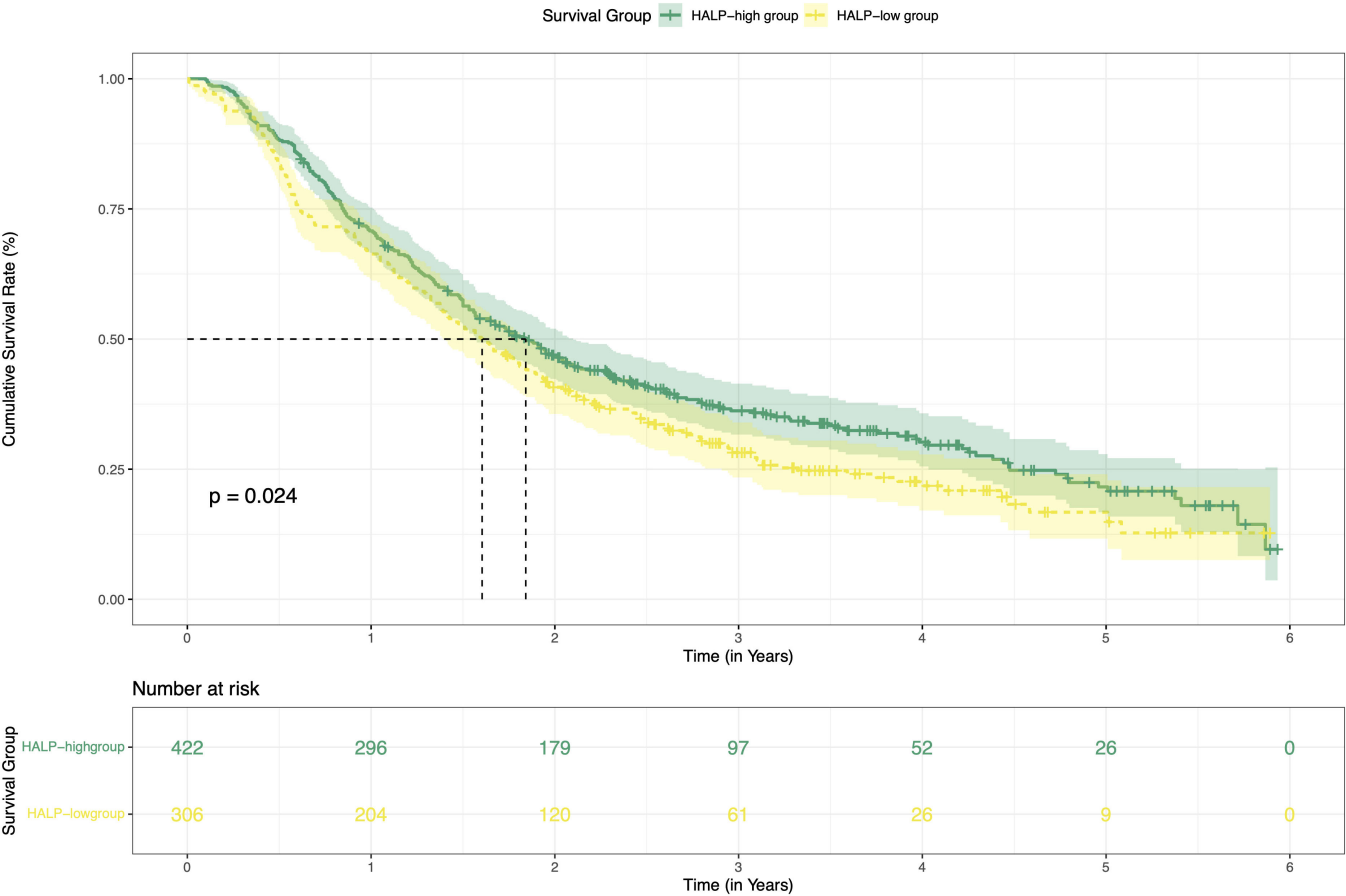
Kaplan-Meier curves of RFS for the H-HALP and L-HALP patients. RFS, recurrence-free survival.

This study enrolled a total of 728 patients with HCC who received TACE combined with ablation at Beijing Youan Hospital. Among these, 422 patients stratified into the H-HALP group and 306 patients into the L-HALP group. Although a statistically significant difference was observed (*P* = 0.024), the median recurrence-free survival (mRFS) was similar for both groups: 1.84 years (95% CI: 1.44-2.04) for the H-HALP group and 1.60 years (95% CI: 1.39-1.89) for the L-HALP group (**Figure 1**). The 1-, 3-, and 5-year RFS rates for the H-HALP group were 70.8%, 36.2%, and 21.5%, respectively, which was only marginally higher than those of the L-HALP group (1-year: 66.3%; 3-year: 28.2%; 5-year: 16.7%). These findings suggest that despite the slightly improved outcomes in the H-HALP group, the prognosis and recurrence patterns still require further exploration. Furthermore, as shown in **Table 1**, both univariable and multivariable Cox regression analyses identified HALP as an independent predictor of RFS (HR: 1.30; 95% CI: 1.09-1.55; *P* = 0.04). Based on these findings, subsequent analyses focused on the subset of patients within the H-HALP group.

**Table 1:** Cox regression for RFS.

Characteristic	Univariable analysis		Multivariable analysis	
	HR (95%CI)	*P* value	HR (95%CI)	*P* value
Age	1.01 (1.00-1.02)	0.272		
Gender (male vs female)	0.84 (0.64-1.10)	0.204		
Cirrhosis (yes vs no)	1.13 (0.85-1.49)	0.411		
Hypertension (yes vs no)	1.06 (0.87-1.30)	0.554		
Antiviral (yes vs no)	0.92 (0.77-1.11)	0.401		
Smoking (yes vs no)	1.10 (0.90-1.34)	0.363		
Drinking (yes vs no)	0.93 (0.76-1.14)	0.462		
Number (multiple vs single)	1.23 (0.95-1.61)	0.12		
Size (>3 cm vs ≤3 cm)	1.11 (0.88-1.40)	0.378		
BCLC				
B vs 0	0.63 (0.44-0.90)	0.011	0.55 (0.43-0.70)	<0.0001
A vs 0	0.48 (0.30-0.78)	0.003	0.38 (0.29-0.50)	<0.0001
WBC	0.98 (0.94-1.03)	0.384		
RBC	0.85 (0.71-1.02)	0.073		
AST	1.00 (0.99-1.01)	0.472		
Glob	1.02 (1.00-1.03)	0.078		
TBIL	1.00 (0.99-1.01)	0.908		
AFP (≥400 vs <400)	1.00 (1.00-1.00)	0.934		
HALP	1.36 (1.12-1.64)	0.002	1.30 (1.09-1.55)	0.004
Diabetes (yes vs no)	1.01 (0.82-1.25)	0.905		
GGT	1.00 (1.00-1.00)	0.001	1.00 (1.00-1.00)	<0.0001

RFS, recurrence-free survival; BCLC, Barcelona Clinic Liver Cancer; WBC, white blood cell; RBC, red blood cell; GGT, γ-glutamyl transpeptidase; ALT, alanine aminotransferase; Glob, globulin; TBIL, total bilirubin; AFP, alpha-fetoprotein.

### Baseline characteristics

Patients in the H-HALP were randomly divided into the training cohort (n=296) and the internal validation cohort (n=126) in a 7:3 ratio. The external validation cohort consisted of 147 individuals. Baseline characteristics are summarized in **Table 2**. In both cohorts, the majority of patients were male (73.6% vs. 74.6% vs. 82.3%) and aged over 55 years old (58.1 ± 8.68 vs. 57.9 ± 8.79 vs. 57.16 ± 7.97). In the training cohort, 259 (87.5%) patients were diagnosed with cirrhosis, 86 (29.1%) with hypertension, and 75 (25.3%) with diabetes mellitus, respectively. In the internal validation cohort, 110 (87.3%) had cirrhosis, 39 (31.0%) had hypertension, 30 (23.8%) had diabetes mellitus. In the external validation cohort, cirrhosis was diagnosed in 135 patients (91.8%), with hypertension and diabetes affecting 36 (24.5%) and 35 (23.8%), respectively. Furthermore, more than half of the patients had received antiviral treatment (59.5% vs. 58.7% vs. 55.8%). In terms of tumor characteristics, most patients had solitary tumor (72.0% vs. 69.8% vs. 68.0%) and tumor size were less than 3cm (63.2% vs. 65.0% vs. 74.1%). Statistical analysis indicated no significant differences between the training and validation cohorts (*P* > 0.05).

**Table 2:** Baseline characteristics.

Characteristics	Training cohort (n=296)	Internal validation cohort (n=126)	External validation cohort (n=147)	*P* value
Age	58.11 ± 8.68	57.89 ± 8.79	57.16± 7.97	0.54
Gender				0.12
Female	78 (26.4)	32 (25.4)	26 (17.7)	
Male	218 (73.6)	94 (74.6)	121 (82.3)	
Cirrhosis				0.35
No	37 (12.5)	16 (12.7)	12 (8.2)	
Yes	259 (87.5)	110 (87.3)	135 (91.8)	
Hypertension				0.46
No	210 (70.9)	87 (69.0)	111 (75.5)	
Yes	86 (29.1)	39 (31.0)	36 (24.5)	
Diabetes				0.91
No	221 (74.7)	96 (76.2)	112 (76.2)	
Yes	75 (25.3)	30 (23.8)	35 (23.8)	
Smoking				0.28
No	181 (61.1)	70 (55.6)	77 (52.4)	
Yes	115 (38.9)	56 (44.4)	70 (47.6)	
Drinking				0.19
No	206 (69.6)	81 (64.3)	90 (61.2)	
Yes	90 (30.4)	45 (35.7)	57 (38.8)	
Antiviral				0.76
No	120 (40.5)	52 (41.3)	65 (44.2)	
Yes	176(59.5)	74 (58.7)	82 (55.8)	
Number				0.68
Single	213 (72.0)	88 (69.8)	100 (68.0)	
Multiple	83 (28.0)	38 (30.2)	47 (32.0)	
Size				0.07
≤3 cm	187 (63.2)	82 (65.1)	109 (74.1)	
>3 cm	109 (36.8)	44 (34.9)	38 (25.9)	
BCLC Stage				0.32
0	85 (28.7)	42 (33.3)	54 (36.7)	
A	164 (55.4)	63 (50.0)	77 (52.4)	
B	47 (15.9)	21 (16.7)	16 (10.9)	
WBC (×10^9^/L)	4.93 ± 2.22	3.36 ± 1.96	5.26 ± 2.10	0.08
RBC (×10^12^/L)	4.01 ± 0.63	4.08 ± 0.60	4.09 ± 0.65	0.34
GGT (U/L)	68.45 ± 68.27	61.59 ± 51.56	75.45 ± 68.71	0.21
AST (U/L)	30.31 ± 14.17	32.16 ± 15.32	30.98 ± 12.07	0.46
Glob (g/L)	28.54 ± 5.23	28.34 ± 5.41	28.57 ± 5.40	0.93
TBIL (μmol/L)	18.78 ± 9.67	20.85 ± 10.22	20.20 ± 10.05	0.10
AFP (ng/mL)	396.45 ± 2106.11	372.71 ± 1085.76	311.33 ± 1385.77	0.89

Values are presented as mean and standard deviation (mean ± SD), or frequency (%).

BCLC, Barcelona Clinic Liver Cancer; WBC, white blood cell; RBC, red blood cell; GGT, γ-glutamyl transpeptidase; ALT, alanine aminotransferase; Glob, globulin; TBIL, total bilirubin; AFP, alpha-fetoprotein.

### Identification of predictive factors

**Figure 2:**
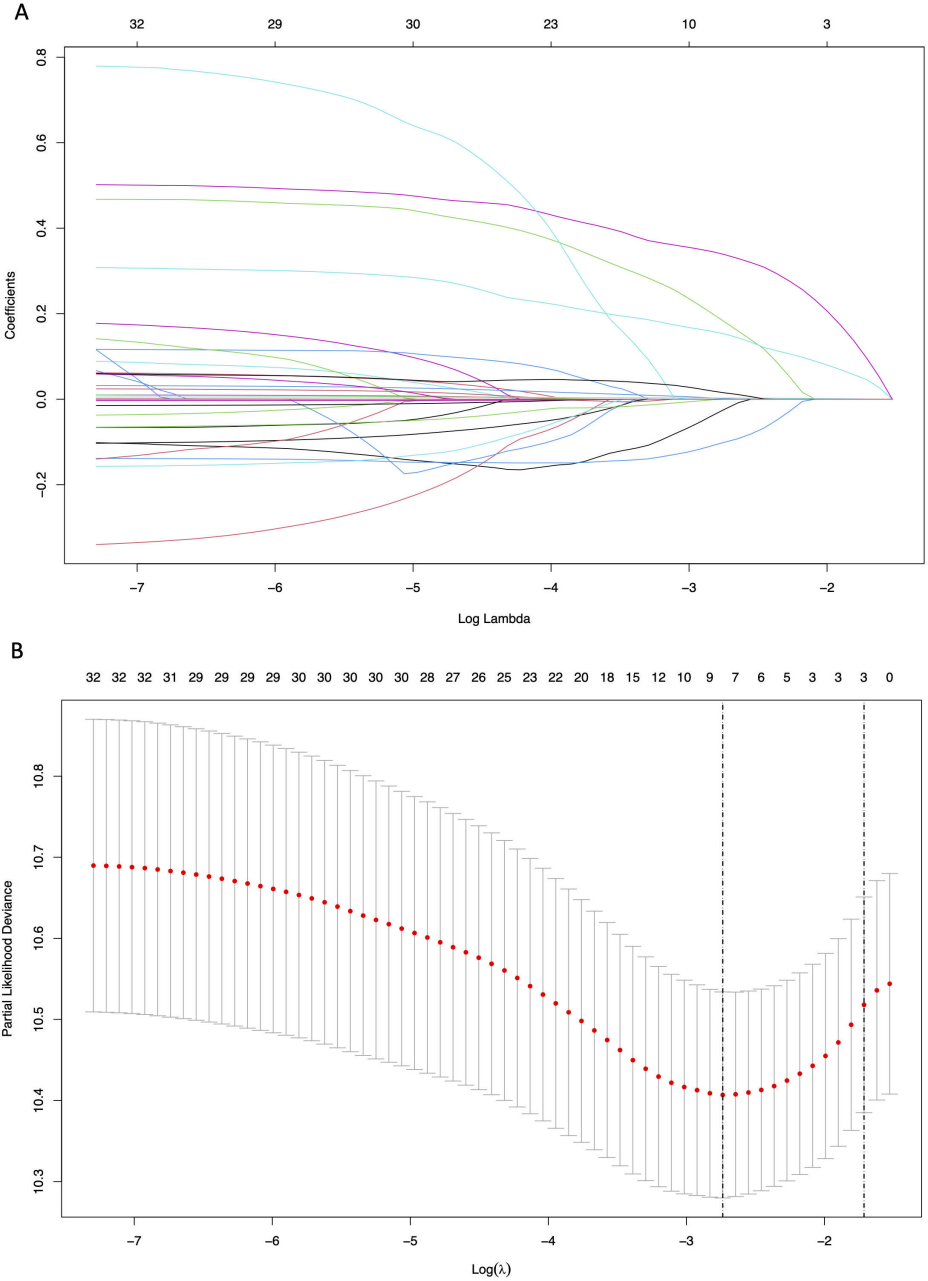
Results of the Lasso regression analysis in the training cohort. **(A)** The variation characteristics of the coefficient of variables; **(B)** The selection process of the optimum value of the parameter λ by cross-validation method.

**Figure 3:**
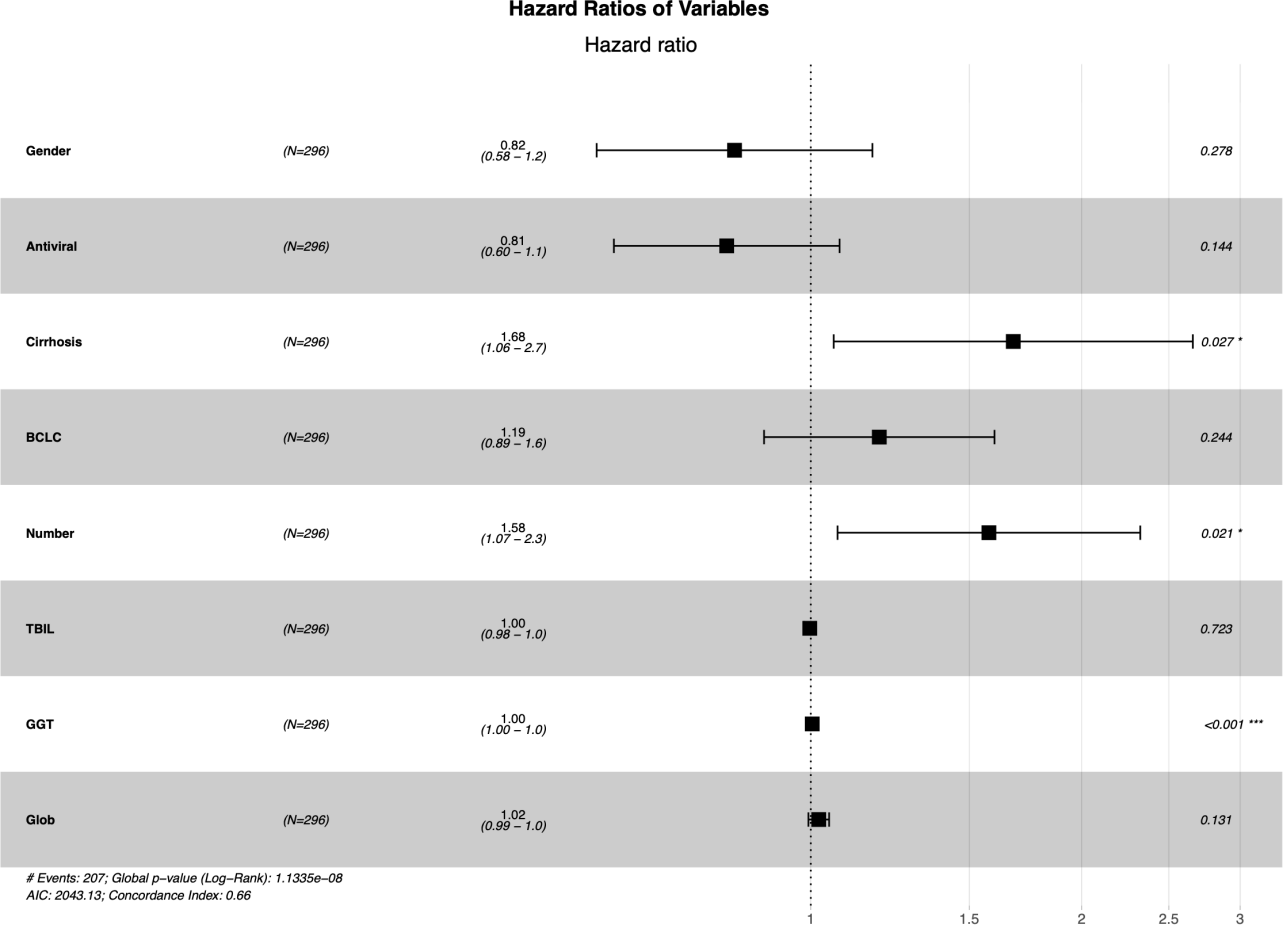
The prediction model with multivariate cox regression.

The LASSO regression analysis was used to screen parameters, and the variation characteristics of the coefficient of these variables were shown in **Figure 2A**. The 10-fold cross-validation method was applied to the iterative analysis, and a model with excellent performance but minimum number of variables was obtained when λ was 0.065 (Log λ = -1.19) (**Figure 2B**). Eight candidate predictors were identified, including gender, antiviral, cirrhosis, BCLC stage, tumor number, TBIL, GGT and Glob (**Figure 3**). These predictors were then assessed through multivariate Cox regression, revealing cirrhosis (*P* = 0.027), multiple tumor (*P* = 0.021) and GGT (*P* < 0.001) were independent predictors of RFS.

### Discrimination performance

**Figure 4:**
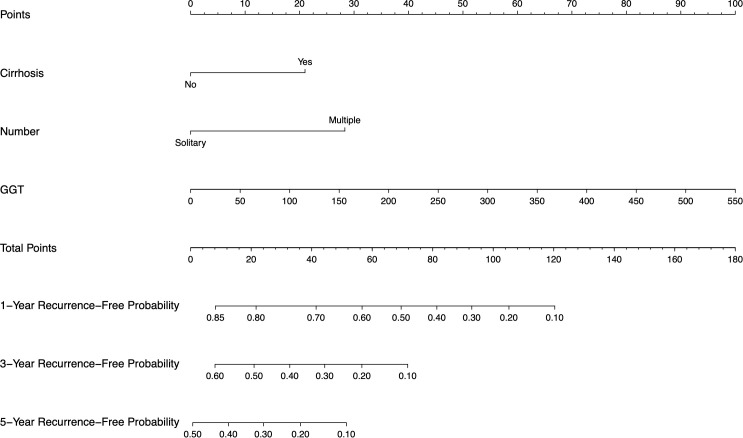
Nomogram, including cirrhosis, tumor number, and GGT for 1-, 3-, and 5-year RFS in HCC patients with high levels of HALP. The nomogram is valued to obtain the probability of 1-, 3-, and 5- years recurrence by adding up the points identified on the points scale for each variable. GGT, γ-glutamyl transpeptidase.

The constructed nomogram incorporated above three screened features identified through multivariate Cox regression (**Figure 4**). To facilitate clinical translation, we developed an interactive web-based version of the nomogram, which is publicly accessible at https://joenomogogogo.shinyapps.io/DynNomapp/. To comprehensively evaluate its discriminatory performance, we compared the nomogram with the AJCC staging system and the individual predictors (cirrhosis, tumor number, and GGT) using time-dependent ROC analysis at 1-, 3-, and 5-year time points. Notably, five-year RFS data were unavailable in the external validation cohort due to insufficient follow-up time in 18.4% of patients. Therefore, 4-year AUC was reported as an alternative endpoint. The nomogram consistently demonstrated superior or comparable AUC values across all cohorts.

**Figure 5:**
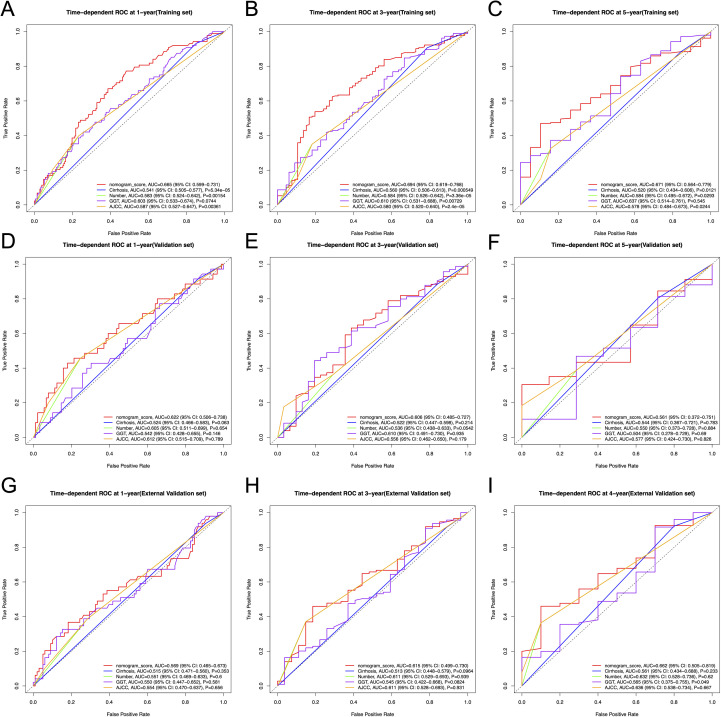
Time-dependent ROC curves for 1-, 3-, and 5-year RFS prediction in the training, internal validation, and external validation cohorts, comparing the nomogram with the AJCC staging system and individual predictors (cirrhosis, tumor number, and GGT). **(A-C)** Time-dependent ROC curves for 1-, 3- and 5-year RFS in the training cohort. **(D-F)** Time-dependent ROC curves for 1-, 3- and 5-year RFS in the internal validation cohort. **(G-I)** Time-dependent ROC curves for 1-, 3- and 4-year RFS in the external validation cohort. ROC, receiver operating characteristic curve; AUC, area under the ROC curve; RFS, recurrence-free survival; AJCC, American Joint Committee on Cancer.

In the training set, the nomogram achieved AUCs of 0.665 (95% CI: 0.599-0.731), 0.694 (95% CI: 0.619-0.768), and 0.671 (95% CI: 0.564-0.779) at 1-, 3-, and 5-year time points, respectively, outperforming the AJCC staging system (AUCs: 0.587 [95% CI: 0.527-0.647], 0.580 [95% CI: 0.520-0.640], and 0.578 [95% CI: 0.484-0.673]; *P* = 0.00361, 2.4e-05, and 0.0244) as well as individual predictors (**Figures 5A-C**). In the internal validation cohort, the nomogram yielded AUCs of 0.622 (95% CI: 0.506-0.738), 0.606 (95% CI: 0.485-0.727), and 0.561 (95% CI: 0.372-0.751) at the same time points, comparable to those of the AJCC system (0.612 [95% CI: 0.515-0.709], 0.556 [95% CI: 0.462-0.650], and 0.577 [95% CI: 0.424-0.730]; *P* = 0.789, 0.179, and 0.826) (**Figures 5D-F**). Similar trends were observed in the external validation cohort, where the nomogram achieved AUCs of 0.569 (95% CI: 0.465-0.673), 0.615 (95% CI: 0.499-0.730), and 0.662 (95% CI: 0.505-0.819) (at 1-, 3-, and 4-year time points), compared with 0.554 (95% CI: 0.470-0.637; *P* = 0.656), 0.611 (95% CI: 0.528-0.693; *P* = 0.931), and 0.636 (95% CI: 0.538-0.734; *P* = 0.667) for the AJCC system (**Figures 5G-I**).

Although the differences in the validation cohorts did not reach statistical significance, likely due to relatively small sample sizes and limited follow-up time, the nomogram demonstrated a consistent trend of improved discrimination across all cohorts.

### Calibration, concordance, and clinical utility

To further evaluate predictive accuracy, we calculated the concordance index (C-index) for each model. In the training set, the nomogram achieved a C-index of 0.646 (95% CI: 0.608-0.684), comparable to AJCC (0.678; 95% CI: 0.615-0.741), while cirrhosis, tumor number, and GGT yielded C-indices of 0.650 (95% CI: 0.542-0.759), 0.692 (95% CI: 0.624-0.759), and 0.579 (95% CI: 0.537-0.621), respectively. In the internal validation cohort, the nomogram’s C-index was 0.614 (95% CI: 0.546-0.683), close to that of AJCC (0.662; 95% CI: 0.565-0.759), and tumor number (0.661; 95% CI: 0.551-0.771), and clearly higher than cirrhosis (0.402; 95% CI: 0.243-0.561) and GGT (0.560; 95% CI: 0.49-0.631). In the external validation cohort, the nomogram attained a C-index of 0.577 (95% CI: 0.519-0.636), similar to AJCC (0.612; 95% CI: 0.517-0.706) and tumor number (0.622; 95% CI: 0.523-0.720), and superior to cirrhosis (0.524; 95% CI: 0.359-0.689) and GGT (0.546; 95% CI: 0.485-0.607). These results suggest that the nomogram offers stable and balanced discriminatory performance across datasets, supporting its potential clinical utility in predicting RFS in patients with HCC undergoing TACE combined with ablation.

**Figure 6:**
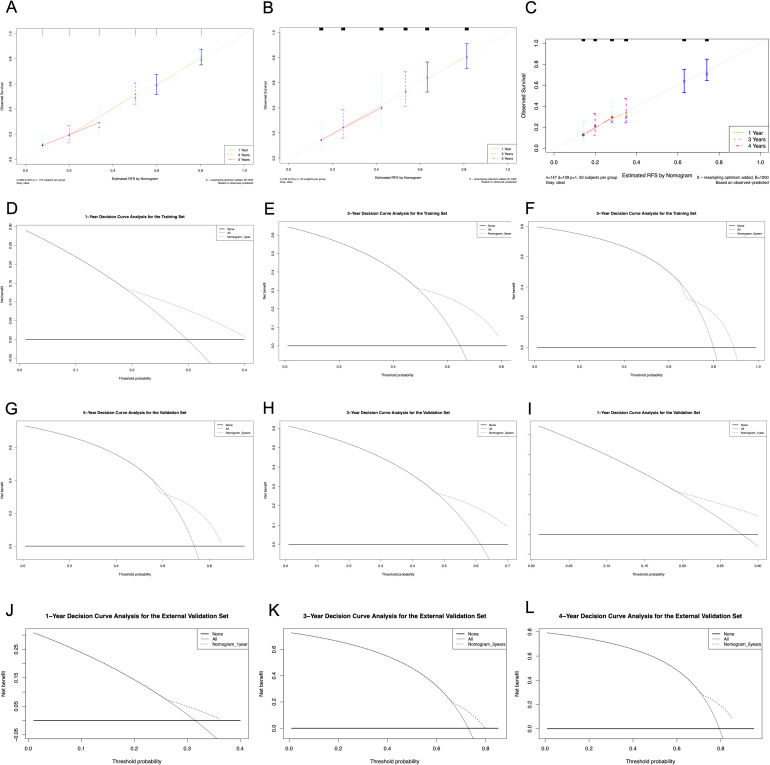
Calibration curves and DCA curves of the nomogram in the training and validation cohort. **(A)** Calibration curves of the training cohort; **(B)** Calibration curves of the internal validation cohort. **(C)** Calibration curves of the external validation cohort. **(D-F)** DCA for 1-, 3- and 5-year RFS in the training cohort. **(G–I)** DCA for 1-, 3- and 5-year RFS in the internal validation cohort. **(J-L)** DCA for 1-, 3- and 4-year RFS in the external validation cohort. DCA, decision curve analysis; RFS, recurrence-free survival.

Furthermore, calibration curves illustrated well agreement between the predicted outcomes and the actual observations (**Figures 6A-C**). And the DCA confirmed the clinical utility of the nomogram, with net benefits consistently exceeding default strategies across risk thresholds (**Figures 6D-L**).

### Risk stratification and survival outcomes

**Figure 7:**
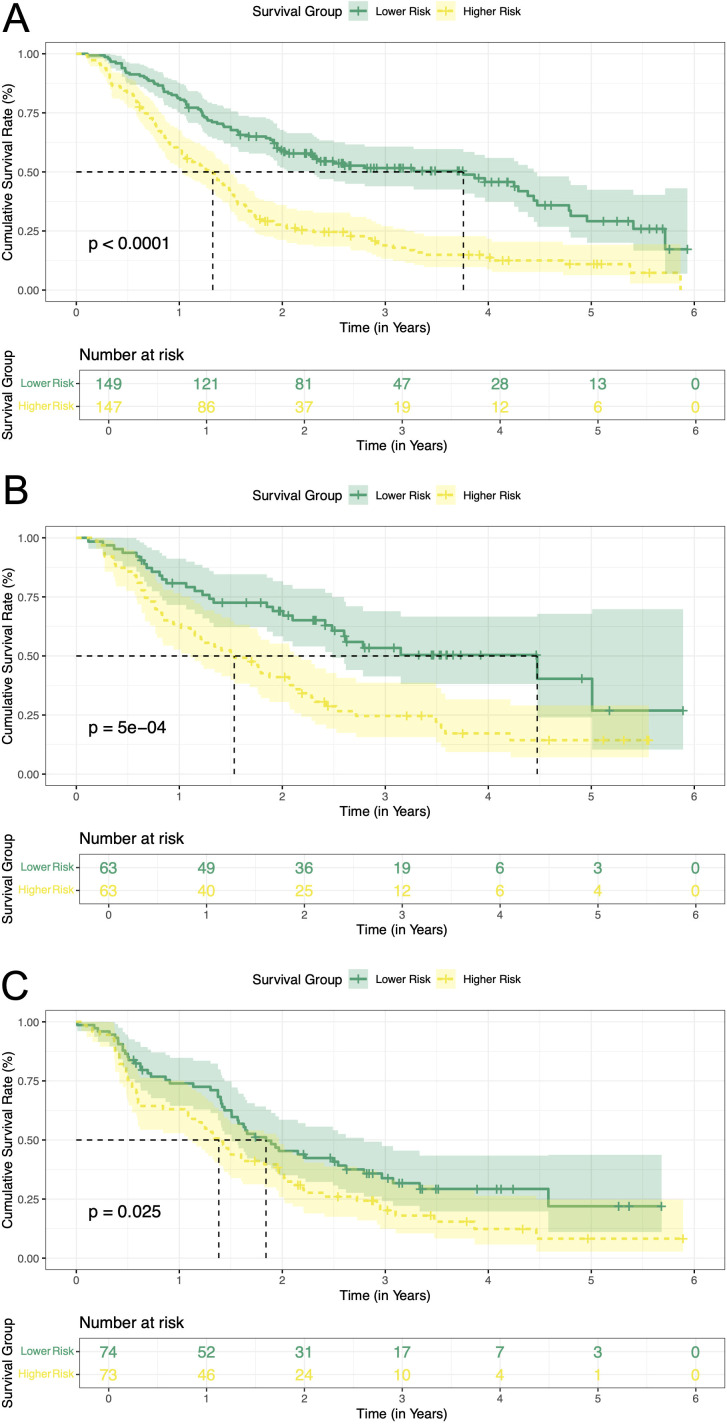
Kaplan-Meier curves of RFS for two risk groups classified by the nomogram in training and validation cohort. **(A)** training cohort; **(B)** internal validation cohort. **(C)** external validation cohort. RFS, recurrence-free survival.

The above analyses demonstrated the good predictive effect of the nomogram. We calculated the prediction score based on the three variables in the nomogram. A median cutoff value was used to separate the patients in the training cohort into a low-risk group (n = 149) and a high-risk group (n = 147). In the training cohort, a significantly prolonged RFS have been observed in the low-risk group (3.76 years, 95% CI: 2.29-4.44) compared with the high-risk group (1.33 years, 95% CI: 1.03-1.50, *P* < 0.0001) (**Figure 7A**). Additionally, the cumulative RFS rates for low-risk patients at 1-, 3-, and 5-year were 0.81, 0.52 and 0.29, respectively, while for high-risk patients, the rates were 0.59, 0.19 and 0.11. In the internal validation cohort, there were 63 patients in each of the low-risk and high-risk groups. The low-risk group achieved a median RFS of 4.48 years (95% CI: 2.48-not reached), with 1-, 3-, and 5-year RFS rates of 0.81, 0.53 and 0.40, respectively. In contrast, the high-risk cohort displayed markedly reduced survival, registering a median RFS of 1.53 years (95% CI: 1.11-2.18, *P* = 5e-04). Corresponding 1-, 3-, and 5-year RFS rates were 0.63, 0.25 and 0.14, respectively (**Figure 7B**). External validation further corroborated this risk stratification pattern. Patients in the low-risk group had significantly longer RFS compared to patients in the high-risk group (1.84 years [95% CI: 1.51-2.79] vs. 1.38 years [95% CI: 1.13-1.97], *P* = 0.025) (**Figure 7C**). The 1-, 3-, and 5-year RFS rates were 0.74, 0.34 and 0.22 in the low-risk patients, while 0.63, 0.20 and 0.08 in the high-risk patients, respectively. All these results proved that our model can effectively distinguish the recurrence risk.

## Discussion

The combination of TACE and ablation has been widely adopted in clinical practice, which offers clear visualization of HCC lesions, expands the ablation zone, reduces tumor volume, and thereby enhancing the complete ablation rate (22). However, it cannot be ignored that a high recurrence rate after therapy. The HALP score, developed by Chen et al. in 2015, provides a comprehensive assessment of both nutritional and immune status (15). Recent studies have identified it as an independent predictor of RFS in pancreatic cancer and early-stage breast cancer, with lower HALP scores associated with shorter RFS (11, 23). Although the prognostic value of the HALP score has been explored in HCC patients undergoing hepatic resection (16–18), its significance in those treated with TACE and ablation remains unclear. Therefore, it is necessary to evaluate the relationship between the HALP score and recurrence in HCC patients treated with TACE and ablation.

Our study indicated that the H-HALP group exhibited a marginally prolonged median RFS compared to the L-HALP group (1.84 vs. 1.60 years, *P* = 0.024). However, the 5-year RFS rates in the high HALP group remained as low as 21.5%, underscoring the limited standalone predictive efficacy of the HALP score for recurrence risk. This aligns with findings by Chen et al. (15) in gastric cancer, where HALP required integration with other indicators to improve predictive power. Notably, the H-HALP patients who defined as “low-risk” by HALP yet experience considerable recurrence rates, need to further risk-stratify in this subgroup. Through Lasso-Cox regression analysis, we developed a nomogram to predict 1-, 3-, and 5-year recurrence of H-HALP patients. This regression method effectively addresses the limitations in overfitting and multicollinearity compared with univariate regression. The nomogram incorporating cirrhosis, tumor multiplicity, and GGT significantly enhanced individualized risk stratification, with marked median RFS disparities between high- and low-risk subgroups across training and validation cohorts.

The HALP score functions as a composite biomarker, reflecting both nutritional depletion and inflammatory activation in HCC. Local inflammation is associated with tumor development and forms part of the tumor microenvironment, while systemic inflammation arises as a response to malignant tumors, mediated by immune proteins, cytokines, and immune cell (24–26). The inflammatory markers such as the neutrophil-to-lymphocyte ratio (NLR) and the platelet-to-lymphocyte ratio (PLR) have demonstrated prognostic value in predicting HCC recurrence (27). Low LYM and elevated PLT levels may indicate compromised immunity and an increased risk of infection (11). LYM are critical to the body’s antitumor immune response. CD4^+^ cells, for instance, enhance this response by promoting the production of antibodies from B lymphocytes and facilitating the differentiation of CD8+ cells, which are responsible for recognizing tumor antigens and directly eliminating cancer cells (28, 29). Additionally, PLT plays a critical role in cancer metastasis by releasing vascular endothelial growth factor (VEGF) and promoting tumor angiogenesis (30). Factors secreted by tumors, such as tumor necrosis factor-alpha (TNF-α) and interleukin-6 (IL-6), can alter the hematopoietic environment, leading to decreased Hb levels (31). Low Hb levels induce tumor hypoxia, activating HIF-1α to promote epithelial-mesenchymal transition (EMT) (32, 33). Meanwhile, low ALB levels reflect hepatic inflammatory status and high nutritional risk, leading to decreased antioxidant capacity and MMP-9 overexpression, further disrupting the extracellular matrix and promoting angiogenesis, both of which contribute to poor oncologic outcomes (34).

The three variables (cirrhosis, tumor number, GGT) that we used to construct prognostic models played important roles in the recurrence and progression of HCC (35–37). Liver function impairment in patients with cirrhosis was a major risk factor for the occurrence of HCC (38). Sasaki et al. (39) found that the recurrence risk in HCC patients with cirrhosis was 6% to 15% higher than those without cirrhosis. Similarly, Jung et al. (40) established a correlation between HCC recurrence and cirrhosis. The scarring caused by cirrhosis compresses intrahepatic blood vessels, impairing oxygen delivery within the liver (41). Consequently, in cirrhotic nodules, the expression of angiogenic factors in hepatocytes is elevated, primarily through the production of hypoxia-inducible factor-1 and other cytokines, which subsequently induce fibrosis and angiogenesis, ultimately leading to portal hypertension and tumor development (42). The presence of multiple tumors is indicative of greater tumor aggressiveness (35). Chan et al. (43) and Xu et al. (44) found 2~3 tumors correlate with a higher recurrence rate than solitary tumor in HCC patients. GGT is an enzyme involved in the metabolism of glutathione and the conversion of γ-glutamyl compounds (45). Elevated GGT levels have been consistently associated with various stages of HCC progression and may serve as a potential biomarker for HCC treatment response (46–49).

Our study also has some limitations. First, as a retrospective analysis, it may introduce selection bias. Second, molecular biomarkers (e.g., ctDNA, PD-L1) were not included due to limited data availability. The HALP score, as a routine blood test parameter, offers advantages of low cost and strong accessibility. Future prospective studies could integrate this nomogram with genomic features (e.g., TP53 mutations, TMB) to further enhance predictive accuracy. Furthermore, the optimal HALP cutoff remains uncertain. Our study consistent with the developers’ choice, using a cutoff value of 56.8 (15). However, some studies have found that the median HALP score varies by cancer type, highlighting the need for further exploration of the heterogeneity of the HALP score (12). Importantly, while we conducted external validation using an independent cohort, all patients were from similar clinical settings in China, which may limit generalizability. Future prospective, multicenter studies with diverse populations are needed to validate our model.

## Conclusion

HCC patients with a high HALP score who underwent TACE combined with ablation had a high recurrence risk. This study developed a nomogram to predict recurrence for these H-HALP patients. For patients identified as high-risk by the nomogram, intensified surveillance (e.g., quarterly imaging) and early initiation of systemic therapy (e.g., TKIs or PD-1 inhibitors) may be considered to mitigate recurrence risk. Integration of the model into clinical decision-making could facilitate risk-adapted therapeutic strategies.

## Data Availability

The raw data supporting the conclusions of this article will be made available by the authors, without undue reservation.
